# Capturing sequence ambiguity among taxa in a primer-specific manner to improve taxonomic classification of amplicon sequencing

**DOI:** 10.1093/nar/gkaf1291

**Published:** 2025-11-29

**Authors:** Jacob T Nearing, Kelsey N Thompson, Thomas Kuntz, Artemis S Louyakis, Amrisha Bhosle, Tobyn Branck, Dayakar V Badri, Eric A Franzosa, Christoph Brockel, Curtis Huttenhower, Meghan I Short

**Affiliations:** Department of Biostatistics, T.H. Chan School of Public Health, Harvard University, Boston, MA 02115, United States; Infectious Disease and Microbiome Program, Broad Institute of MIT and Harvard, Cambridge, MA 02142, United States; The Harvard Chan Microbiome in Public Health Center, T.H. Chan School of Public Health, Harvard University, Boston, MA 02115, United States; Department of Biostatistics, T.H. Chan School of Public Health, Harvard University, Boston, MA 02115, United States; Infectious Disease and Microbiome Program, Broad Institute of MIT and Harvard, Cambridge, MA 02142, United States; The Harvard Chan Microbiome in Public Health Center, T.H. Chan School of Public Health, Harvard University, Boston, MA 02115, United States; Department of Biostatistics, T.H. Chan School of Public Health, Harvard University, Boston, MA 02115, United States; Infectious Disease and Microbiome Program, Broad Institute of MIT and Harvard, Cambridge, MA 02142, United States; The Harvard Chan Microbiome in Public Health Center, T.H. Chan School of Public Health, Harvard University, Boston, MA 02115, United States; Hill’s Pet Nutrition, Inc, Topeka, KS 66617, United States; Department of Biostatistics, T.H. Chan School of Public Health, Harvard University, Boston, MA 02115, United States; Infectious Disease and Microbiome Program, Broad Institute of MIT and Harvard, Cambridge, MA 02142, United States; The Harvard Chan Microbiome in Public Health Center, T.H. Chan School of Public Health, Harvard University, Boston, MA 02115, United States; Department of Biostatistics, T.H. Chan School of Public Health, Harvard University, Boston, MA 02115, United States; The Harvard Chan Microbiome in Public Health Center, T.H. Chan School of Public Health, Harvard University, Boston, MA 02115, United States; Hill’s Pet Nutrition, Inc, Topeka, KS 66617, United States; Department of Biostatistics, T.H. Chan School of Public Health, Harvard University, Boston, MA 02115, United States; Infectious Disease and Microbiome Program, Broad Institute of MIT and Harvard, Cambridge, MA 02142, United States; The Harvard Chan Microbiome in Public Health Center, T.H. Chan School of Public Health, Harvard University, Boston, MA 02115, United States; Hill’s Pet Nutrition, Inc, Topeka, KS 66617, United States; Department of Biostatistics, T.H. Chan School of Public Health, Harvard University, Boston, MA 02115, United States; Infectious Disease and Microbiome Program, Broad Institute of MIT and Harvard, Cambridge, MA 02142, United States; The Harvard Chan Microbiome in Public Health Center, T.H. Chan School of Public Health, Harvard University, Boston, MA 02115, United States; Department of Immunology and Infectious Diseases, T.H. Chan School of Public Health, Harvard University, Boston, MA 02115, United States; Department of Biostatistics, T.H. Chan School of Public Health, Harvard University, Boston, MA 02115, United States; The Harvard Chan Microbiome in Public Health Center, T.H. Chan School of Public Health, Harvard University, Boston, MA 02115, United States; Institute for Clinical Research and Health Policy Studies, Tufts Medical Center, Boston, MA 01803, United States; Department of Medicine, Tufts University School of Medicine, Boston, MA 01803, United States

## Abstract

Amplicon sequencing, a common strategy to taxonomically profile microbial communities, is relatively low cost and high throughput. However, it is subject to unique biases, including primer incompatibilities and the inability to differentiate between certain microbes due to low sequence variability. Due to this, taxa may be mis-, multiply-, or un-identified when using different variable regions. To address this, we developed Parathaa (Preserving and Assimilating Region-specific Ambiguities in Taxonomic Hierarchical Assignments for Amplicons), which directly models taxonomic sequence ambiguities within amplicon regions and allows for assignments to multiple taxonomic labels when phylogenetically warranted. Parathaa accomplishes this by leveraging full-length sequence databases to build primer-specific phylogenies, which it uses to identify variable-region-specific taxonomic distance thresholds. Parathaa then assigns taxonomy to sequences by placing them into these trees, allowing for multiple assignments if the tree is not resolved at the placement location. Thus, Parathaa’s assignments capture biological ambiguities specific to the sequenced variable region. Parathaa performed better than both IDTAXA and RDP-based Naïve Bayes classifiers with or without exact matching (as implemented in DADA2) at the species level when applied to a synthetic dataset from across the bacterial kingdom. Overall, Parathaa’s approach allows users to retain more information and understand potential sources of bias when classifying amplicon reads

## Graphical Abstract

**Graphical Abstract:**
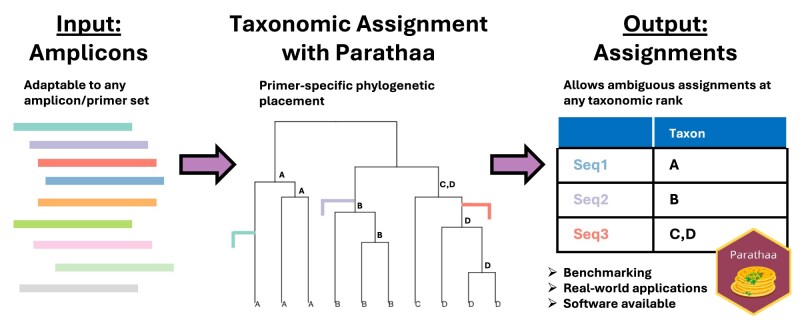

## Introduction

Amplicon sequencing is widely used for taxonomic characterization of microbial samples. This relatively low-cost and high-throughput method involves the polymerase chain reaction amplification of hypervariable regions of a well-conserved marker gene, such as the 16S ribosomal RNA (rRNA) or cpn60 gene in prokaryotes [1], the 18S rRNA gene in eukaryotes [2], or the ITS gene region in fungi [3]. The taxonomic level to which confident assignments can be made varies widely [4–6], and as with any sequencing method, amplicon sequencing is subject to several inherent biases [7, 8]. As a result, amplicon sequences are often either incorrectly assigned to a single taxon—ignoring other, potentially identical matches—or left unassigned to an optimally specific (e.g. species) level.

**Figure 1.**
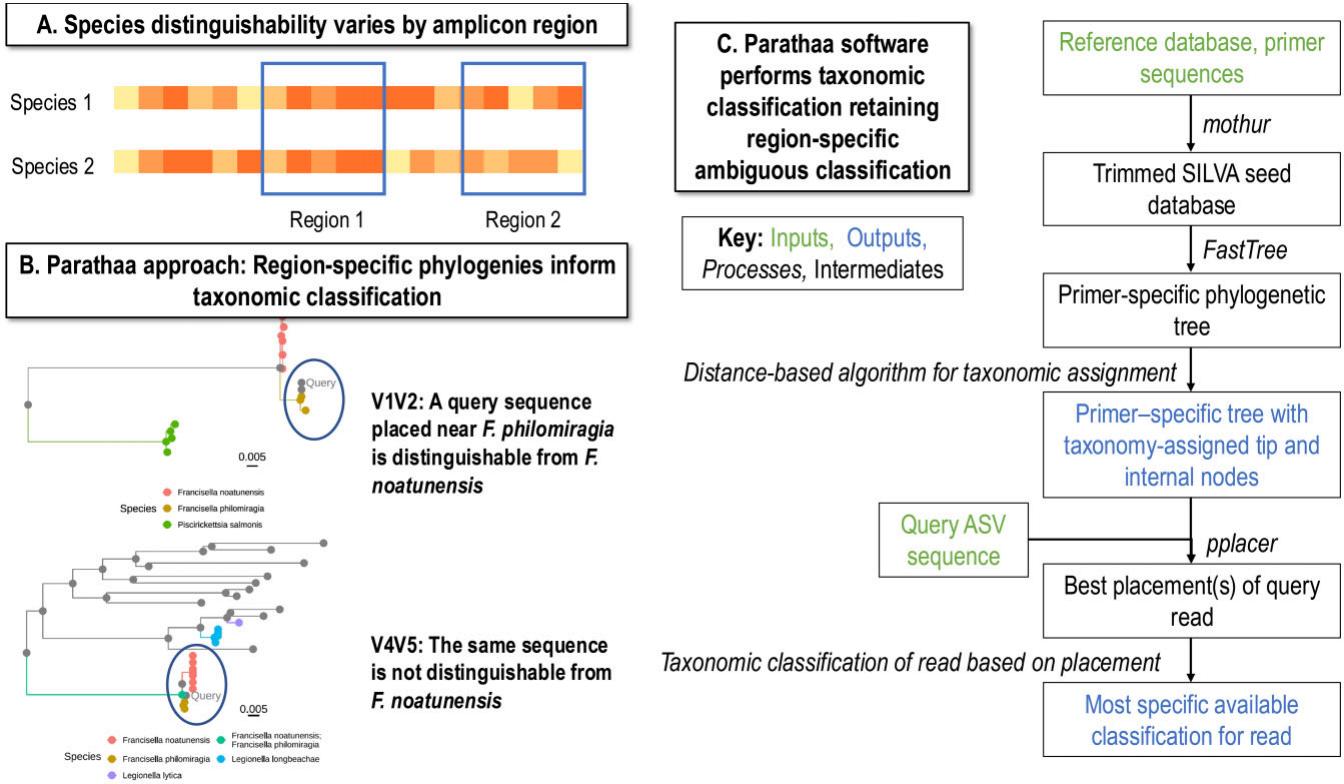
Primer-specific, phylogenetically informed taxonomic assignment of amplicons by Parathaa. **(A)** Depending on an investigator’s selection of marker gene hypervariable regions for amplicon sequencing, certain species (or higher-level clades) will be uniquely identifiable within that region while others will not. **(B)** To optimize taxonomic classification with respect to phylogenetic ambiguity, Parathaa constructs region-specific phylogenies and propagates taxonomic classifications to interior nodes using region-optimized taxonomic distance thresholds. Query sequences are classified by being placed into the tree and assigned taxonomy via proximal nodes. **(C)** Workflow for primer-specific taxonomic assignments of amplicons with Parathaa. Inputs in green are provided by the user. Reference databases should be aligned sequences of the amplicon of interest (i.e. 16S rRNA genes, ITS regions etc).

These limitations are particularly applicable to short-read sequencing technologies, which account for the vast majority of amplicon studies to date. Amplicons of only a few hundred bases only sequence subsets of marker genes, or more specifically, one to three (typically) hypervariable regions of these genes. Choice of the specific region to be sequenced leads to primer- and region-specific biases that affect performance of taxonomic classification (Fig. 1A) [9]. These biases include (i) differential primer annealing leading to biases in amplification, as in the observed problems quantifying *Bifidobacteriales* using the V1V2 region of the 16S rRNA gene due to lack of binding of the 27F primer [10], and (ii) sequence specificity (i.e. differentiability) varying by hypervariable region [11]. Because of the sequence variability bias in particular, some taxa can be classified to lower taxonomic levels than others depending on the region sequenced. For example, species in the *Staphylococcus* genera have low entropy in the V4 region of the 16S rRNA gene, making it difficult to speciate them using a V4 region primer set [12]. While less common, such limitations can also apply to longer-read sequencing, generally due to database incompleteness (e.g. for fungal ITS region) [6].

In either setting, existing taxonomic classifiers generally assign taxonomy to the lowest level at which there is sufficient confidence, while optionally giving multiple top “hits” akin to BLAST + or vsearch [13, 14]. However, typically a single assignment is used at a given taxonomic rank, or the sequence read remains unassigned at that rank. This is the case even if there is high confidence that a sequence belongs to one of a small subset of members of a lower rank. For example, a sequence could be assignable to a subset of three species within a 20 species genus, yet most classification tools will only assign a genus rank. Amplicon classifiers that do operate at the species level often do so conservatively, as a result, assigning either very few species [15] or, alternatively, incurring substantial false positive rates [16]. For these reasons, researchers often default to analyzing amplicon data at the genus level—a compromise that can often hinder the ability to mechanistically investigate the contributions of individual microbes.

Here, we introduce Parathaa (Preserving and Assimilating Region-specific Ambiguities in Taxonomic Hierarchical Assignments for Amplicons), a method of assigning taxonomy to amplicon sequences that, as needed, preserves one-to-multiple mappings of reads to taxonomic classifications at a given level, taking into account the primer-specific regional ambiguity present when using short read sequencing. By doing so, Parathaa retains more information about the taxonomy of reads and directly models potential region-specific ambiguities within amplicons of interest. Overall, we aim to increase the information obtainable from short-read amplicon sequencing and to provide a consistent framework to predict identifiable taxa for various primer pairs/gene regions. Parathaa accomplishes this by leveraging existing 16S rRNA gene sequence databases to build primer-specific phylogenies, followed by taxonomic assignments that capture the ambiguities specific to the phylogeny-taxonomy correspondence at that primer-pair-specified region. While we focus on the 16S rRNA gene for bacterial taxonomic classification, the method is designed to be gene-, region-, and reference-independent (Fig. 1B and C). Furthermore, Parathaa allows users to plot the regional tree where a sequence is placed, intrinsically providing assignment context not given by e.g. Naïve Bayes methods (Fig. 1B). We applied and evaluated Parathaa using (i) five synthetic read datasets from SILVA sequences external to Parathaa’s database, (ii) newly added sequences to GTDB R220, after training on GTDB R202, (iii) reads from a published culture-based mock community [8], and (iv) sequences from both human oral [17] and mine sediment (environmental) microbial communities.

## Methods

### Parathaa method overview

Broadly, Parathaa assigns taxonomy while retaining primer-specific ambiguities at each taxonomic level by (i) creating a primer-pair-specific reference phylogeny, (ii) propagating taxonomies from the reference sequence tips to interior nodes of the tree (retaining one-to-many ambiguous assignments where warranted), (iii) placing sequences to be identified into the phylogenetic tree, and (iv) assigning the taxonomy of the proximal node. The relatively computationally expensive steps (i) and (ii) are only needed once per database and primer pair; any number of samples can be identified relatively quickly once these steps are complete. Additionally, pre-computed trees are available for commonly used 16S rRNA gene regions (V1V2, V1V3, V3V4, V4V5) and full length 16S rRNA gene sequences using the Silva 132 seed database, and GTDB R220 database.

### Parathaa method details

Parathaa’s first step is to create a primer-specific phylogeny. We used a seed database based on SILVA version 138.1[18] distributed by mothur. This comprises a subset of the entire Silva alignment file that selects sequences that match with 100% identity to those found in the Silva SEED database. However, it is possible to use other databases of marker genes with corresponding taxonomies within Parathaa. Full-length 16S rRNA gene sequences from the input database are trimmed to a particular amplicon region based on an input file of primer sequences using mothur (version 1.48.0) [19]. The phylogeny is built from the trimmed sequences using FastTree (version 2.1.11) [20].

When the tree is built, only the tip nodes have an assigned taxonomy, which comes from the taxonomy of the original reference sequences. Minor curation of the sequence labels is performed, such that any subspecies-level information is grouped to the species level, and any unclassified sequences are given missing designations. Subsequently, Parathaa propagates taxonomy up through the tree as follows. First, optimal cophenetic distance thresholds based on the general time reversible model of nucleotide evolution are identified, which will define taxonomic groups at each level. For instance, any node whose underlying tips have pairwise distances less than the genus-defining threshold are considered to be of the same genus. To find the optimal thresholds at each taxonomic level, a threshold-finding step tries a range of distance cutoffs for each taxonomic level, and calculates an associated error based on misclassification of the known sequences. The chosen threshold is that which minimizes (i) grouping tips of multiple ground-truth taxa under a single taxon-defining node (“over-merging”), and (ii) splitting of sequences from the same ground-truth taxonomic group into multiple taxon-defining nodes (“over-splitting”).

**Figure 2.**
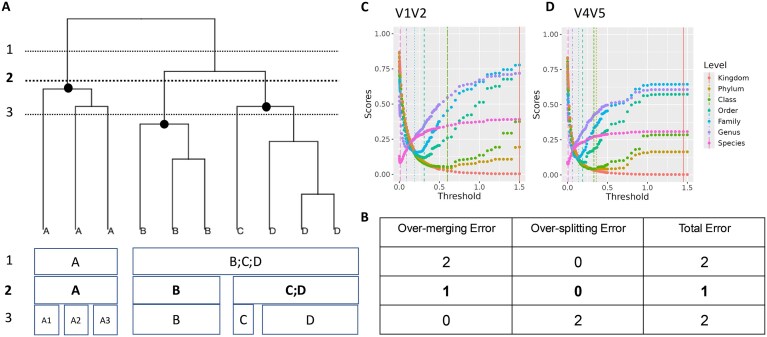
Definition of taxonomic groups with Parathaa; illustration and application to V1V2 and V4V5 subregions. **(A)** Example tree with three possible distance-based thresholds and corresponding taxonomic groups below. Tip taxonomies are known (labeled A–D) and are grouped together based on candidate distance thresholds 1, 2, and 3 in a primer-specific phylogeny. The thresholds are evaluated based on their ability to distinguish sequences of different groups while not splitting sequences of the same group. Based on these criteria, threshold 2 is the optimal choice here, which can be seen in the error table in panel (B). **(B)** Table of over-merging and over-splitting errors, which sum to the total error. **(C, D)** Optimal cophenetic distance thresholds for each taxonomic level for **(C)** V1V2 and **(D)** V4V5 regions of SILVA seed database.

To calculate the over-merging errors for a given threshold, the tree is cut into groups based on the threshold, such that the maximum distance among tips in a group is less than the threshold (Fig. 2A). These groups are temporarily named based on the name of the majority taxon within that group. Any ground-truth taxa other than the majority taxon in that group contributes to the over-merging error (one for each additional taxon represented) (Fig. 2B). To calculate the over-splitting error, all groups across the tree, at the given taxonomic level are tabulated based on their temporary names (i.e. majority taxon). For each duplicate instance of a majority taxon, Parathaa adds one to the over-splitting error, since in the ideal case we expect there to be only one taxonomic group with a given taxonomic label (Fig. 2B). Both error scores are then summed and normalized to a score between 0 and 1 to create an overall tree-wise error rate for that distance threshold. The threshold value at each taxonomic level that minimizes the summed error across the tree is carried forward to the taxonomy assignment step.

Once optimal thresholds for each taxonomic level are identified for a region-specific tree (Fig. 2C and D), taxonomy is assigned to internal nodes of the tree. For each internal node of the tree Parathaa identifies whether the maximum distance among tips under the node is less than the distance threshold for each taxonomic level starting with Phylum and descending to Species. If the node is within the level tested, the names of the underlying tips at that level are collected and the node is assigned taxonomy according to the dominant name. The dominant taxon name is determined through the use of a binomial probability model assuming a 5% error rate in taxonomic classification and permitting a probability of up to 0.20 that a nondominant taxonomic classification was assigned in error. If there is a mix of taxonomic labels of leaf sequences in a taxonomic group such that multiple taxa exceed the error threshold, the taxon is named for all such labels; if no taxon passes the error model all potential taxon names are assigned to the internal node. Once an ambiguous assignment is made, all subsequent assignments are done in a hierarchical fashion by passing the underlying taxon names from each higher level ambiguous taxonomy into the binomial error model separately. This is one of the ways Parathaa achieves the ability to assign multiple taxonomic labels to a single sequence.

New query reads are assigned taxonomic classifications based on placement within the reference tree. Query reads are first aligned against the template input-primer-trimmed reference alignment (from above) using mothur with the command *align.seqs*. Briefly, this command aligns sequences by first searching the template alignment for candidate matches to the query using a kmer search. Once a candidate is found, the query sequence is aligned to the template using Needleman–Wunsch alignment [21] and gaps are filled in the multiple sequence alignment using the NAST algorithm [22]. Finally, the output alignment is then quality controlled to remove query sequences that do not have 80% coverage against their identified template sequence. Reads are placed using the maximum likelihood phylogenetic placement tool *pplacer* (version v1.1.alpha19-0-g807f6f3) [23]. Taxonomy for the new read is assigned with the taxonomic classification of the child node to where the sequence is placed (i.e. the “index node”), to the lowest taxonomic level for which the query is within the taxonomic threshold of all children of the index node. In the case of multiple high-likelihood placements provided by pplacer, placements with likelihood weight ratio within 50% of the greatest value were considered. Of those, the union of taxa at the placements was taken to be the assignment; this is a second mechanism by which multiple taxonomic assignments are made. If the placements disagree regarding appropriate level of classification (e.g. two high-likelihood placements suggest the sequence can be classified at the species level and another suggests it can only be classified to the genus level), the mode was taken to be the appropriate level.

Accurate species-level assignment in Parathaa requires a few additional considerations. We have found that, for the Species level in particular, the “optimal” threshold identified by the algorithm tends to be too large when false positives outweigh the value of true positives. As such we implement an additional parameter to multiply by the Species-level threshold. In Parathaa’s default specific mode, this parameter is set to 0.1. In Parathaa’s sensitive mode this parameter is ignored, and the original Species-level threshold is used.

### Benchmarking against other taxonomic classifiers

To compare Parathaa against other taxonomic classifiers, we benchmarked both IDTAXA and several variants of the RDP Naïve Bayes classifier [24] at the species level (as used by DADA2) using both synthetic and real-world data. All classifiers were trained using the same Silva v138 seed database or GTDB R202 database.

We retrained the IDTAXA classifier on the same database used by other parts of our evaluation, as outlined in the DECIPHER vignette (version 3.0.0) [25]. In brief, we first subset the Silva seed database to those sequences that had terminal species labels (as required by IDTAXA) and then pruned sequences that were represented by >10 species. Next, we trained the IDTAXA classifier over 10 iterations using the learnTaxa() function with default parameters to minimize the number of problematic sequences identified during the training process. We then classified our synthetic datasets using the function IdTaxa(), the previously trained classifier, “type = extended”, and “strand = top” as outlined in the DECIPHER R package vignette.

The RDP-based classifier variants were all benchmarked using the implementation in the R package DADA2 (version 4.4.1), a commonly used library for amplicon data processing. Specifically, we assessed three different Naïve Bayes variants. Two as implemented in DADA2, which initially use the function assignTaxonomy() down to the genus level and then assign species using the addSpecies() function which identifies exact sequence matches between query reads and the reference database. The addSpecies() function also allows users to include cases where a query exactly matches multiple reference sequencings by setting “allowMultiple = TRUE. In addition to these two classifiers, we also included a Naïve Bayes classifier as implemented in RDP using the assignTaxonomy() function (minimum bootstrap = 80) for full length sequences.

All sequences were identically processed as described in the below sections, prior to classification with Parathaa or any of the above classifiers.

### Creation of synthetic reads for benchmarking

Parathaa’s taxonomic assignments come from a subset of the SILVA 16S rRNA gene database (released by *mothur*) [18, 19]. As such, there are SILVA sequences external to Parathaa’s database that can be used to assess the tool’s performance. In the baseline dataset that was used to optimize Parathaa’s sensitive versus specific settings, we randomly selected a subset of external (outside of Parathaa’s database) SILVA sequences with known taxonomic classification to the species level. Sequences were selected so that each bacterial genus was sampled at 1%, or at least five sequences (or all available, if fewer than five sequences were available for a genus). We only included sequences from Genera that were represented in the seed database. We trimmed the sequences to the V1V2 (27F, 338R primers) and V4V5 (515F-Y, 926R primers) regions using the *pcr.seqs* function in mothur to create synthetic ASVs; 11 912 in V1V2 11 667 in V4V5. We removed sequences with unidentified (“N”) bases, resulting in a total of 11 285 (V1V2)/11 349 (V4V5) sequences. We then ran the resulting synthetic ASVs and full length sequences through the Parathaa, IDTAXA, and various Naïve Bayes classifiers, and compared the assigned taxonomies to the ground truth taxonomy. We calculated classification accuracy, precision, recall, and F1 score for each workflow and amplicon set where one-to-many assignments were considered correct if at least one truth member was present, and nonassignments were correct if the ground truth didn’t exist in the database used for classification. A second holdout dataset (“baseline holdout”) was constructed in the same manner as above while not including those that were in the baseline synthetic dataset. This dataset included 8160 V1V2, 8450 V4V5, and 9007 full length sequences.

To understand Parathaa’s performance in random holdout data, we created three additional synthetic sequence datasets. The three hold-out datasets were created from 10 000 random sequences that were both external to Parathaa’s database and those sequences used in the baseline benchmarking dataset.

We also constructed additional synthetic datasets to present more challenging assignment cases. The “single representative genus” dataset was constructed so that one sequence was randomly selected from each genus that was present within the SILVA database (*n* = 3063 sequences) while still excluding those sequences that were in Parathaa’s database. Finally, the “genus outside of DB” dataset was created from 10 000 sequences that were from genera that were not represented in Parathaa’s database thus presenting Parathaa with sequences that it should not assign a taxonomic label to at the genus or species level. Each of these synthetic datasets were processed in the same way to generate V1V2, V4V5, and full length datasets.

### Creation of precision–recall curves

Parameter spaces within the Naïve Bayes classifier and Parathaa were explored through the use of precision–recall curves on a single holdout dataset by varying either the minimum number of bootstraps in the Naïve Bayes classifier or the species distance threshold in Parathaa. For Parathaa, we tested species distance threshold multipliers from 0 to 1.95. In the case of the Naïve Bayes classifier, we calculated the precision and recall values for assignments with minimum bootstrap values from 0 to 99 by sequentially increasing the minimum bootstrapping by 3 over 30 subsequent runs. As the Naïve Bayes exact species matching (Naïve Bayes – Exact) and Naïve Bayes multiple species exact match (Naïve Bayes – Multi) require exact sequence matches at the species level there was no parameter space to explore at the species level.

### Mock community data and datasets for benchmarking

Mock community sequences were obtained from NCBI (SRX1631783, SRX1631785) [8]. We downloaded pre-processed merged paired-end Illumina reads (V1V2: *n* = 540 991 reads; V4V5 *n* = 42 703 reads) from the DNA-based mock communities sequenced on the Illumina MiSeq platform. Along with the previous quality filtering performed by the original authors we also removed reads with ambiguous bases and taxon assignments that were represented by fewer than 30 reads. Raw data from the oral dataset are available on NCBI (PRJEB38175). Raw reads were processed using deblur [27] following the same methods as reported in the data’s original publication [17]. Raw sequence data from the sediment dataset is available on NCBI (PRJNA1167286). Data were processed using QIIME2 (v. 2023.5) [28]. Primers were trimmed using the QIIME2 plugin cutadapt [29] and ASVs were generated using DADA2 with forward read length of 270 bp and a reverse read length of 210 bp. The maximum expected errors for front reads was set to 2 and the reverse reads were set to 3.

## Results

### Parathaa assigns amplicon sequences to its highest known taxonomic level while maintaining sufficient accuracy

Parathaa carries out two overall steps to taxonomically profile amplicon sequences: pre-computing an optimized primer-specific mapping between phylogenetic placement and taxonomy, and applying this to assign zero or more taxonomic labels to each amplicon sequence (Fig. 1C). Prior to analyzing amplicon sequences themselves, given an input primer pair, the first step begins with a full-length marker gene sequence database and trims it to the appropriate region based on primer sequences. These trimmed reference sequences are used to create a region-specific phylogeny for which the taxonomy of the tip sequences is known (see the ‘Methods’ section). Using an algorithm that determines optimal taxonomic distance thresholds (detailed in the ‘Methods’ section), taxonomy of the tips is propagated to the interior nodes of the tree, using distance-based thresholds that are optimized across the (still primer-specific) phylogeny to define taxa at each level (species, genus, family, etc.) For cases where Parathaa-defined taxonomic groups contain reference sequences from multiple taxa, one-to-many taxonomic assignments are given to the relevant interior node(s). In the second step, each amplicon sequence to be classified is placed into the tree via maximum likelihood [23] and taxonomic classification is given based on the most proximal node(s) to this placement (Fig. 1C). Finally, the specificity and sensitivity of Parathaa’s assignments (particularly species) can be adjusted using either two different pre-configured modes [*specific* (default) and *sensitive*] or customizable parameter tuning through settings such as threshold multipliers.

### Parathaa accurately and specifically classifies synthetically held-out amplicon sequences

**Figure 3.**
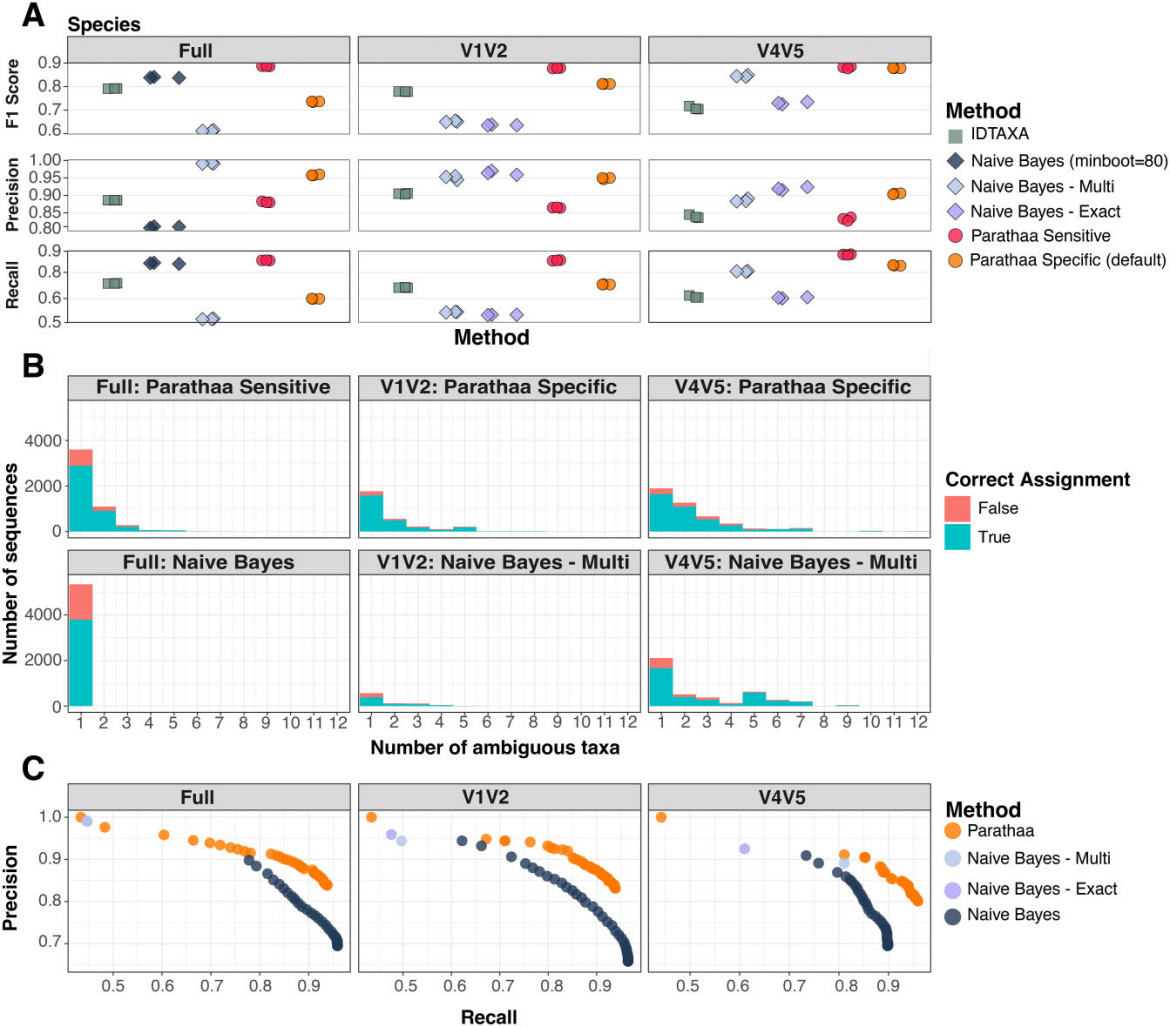
Parathaa performs comparably to or better than other amplicon classifiers on synthetic holdout amplicons. **(A)** Parathaa’s performance was compared to the RDP-based Naïve bayes classifiers and to IDTAXA on both full length 16S rRNA gene sequences and on simulated short hypervariable region amplicons. On short variable regions, RDP Naïve Bayes classifiers were run using default settings while requiring exact sequence matching at the species level (as implemented in DADA2 – Naïve Bayes – Exact and Naïve Bayes – Multi, respectively). In the case of Naïve Bayes – Multi, we also allowed multiple species assignments for each single read. On full length sequences, we evaluated both of these Naïve Bayes classifier variants along with a Naïve Bayes classifier that assigned species without exact matching (but a minimum bootstrapping of 0.80, labelled Naïve Bayes). Reads with ground truth species that were not included in the seed database used by all classifiers were considered true positives if left unassigned. **(B)** Sequences that were assigned taxonomy at the species level on one of the holdout datasets using either Parathaa or the Naïve Bayes variants described above, indicating correct and incorrect taxonomic assignments. The *x*-axis indicates the number of taxa assigned to each sequence; for assignments with multiple taxa, the assignment is considered correct if at least one assigned taxon matches the ground truth. **(C)** Precision–recall curves generated by varying Naïve Bayes or Parathaa parameters for a single holdout dataset (minimum bootstrapping level or species threshold multiplier, respectively). Naïve Bayes – Exact and Naïve Bayes – Multi show the same data as panel (A) and are included as reference points.

To quantify Parathaa’s accuracy relative to a known ground truth, we applied the method to classify reads from seven different synthetic datasets (three of which are replicate holdouts) using full length reads or short reads from the V4V5 or V1V2 16S rRNA gene region (see the ‘Methods’ section). Initial *sensitive* (species threshold multiplier of 1) and *specific* (species threshold multiplier of 0.1) settings were optimized on a benchmark dataset, which consisted of 12 418 synthetic reads drawn from the SILVA database that are external to the seed database used by Parathaa for classification (“baseline”). Reads were from genera included in Parathaa’s database, but not necessarily from species in the database, so that Parathaa’s performance could be assessed on known and novel species. We further compared Parathaa’s performance using three holdout datasets composed of 10 000 sequences each that were selected randomly from the SILVA database (Fig. 3A and Supplemental Fig. 1), excluding those included within the baseline dataset and Parathaa’s seed database.

Finally, three additional datasets were tested: one with reads randomly selected from a single representative of each genus in SILVA (“Single representative genus”), one with reads from genera absent in Parathaa’s default seed database (“Genus outside of DB”), and one additional holdout dataset (“baseline holdout”) that used the same selection parameters as the “baseline” dataset while excluding sequences that were included in “baseline” (Supplemental Fig. 2). Collectively, these synthetic datasets aimed to evaluate Parathaa’s performance in a range of challenging read assignments. We assigned the recall, precision, and F1 score along with the number of sequences that were left unassigned correctly and unassigned incorrectly. Sequences without assignments were considered “unassigned correctly” (true positives) when the taxa (species, genus etc.) that they originated from were not present in the underlying trained database. We compared Parathaa to IDTAXA and the RDP Naïve Bayes classifier [24] (as implemented in DADA2 [26]) using various suggested parameter settings (see the ‘Methods’ section) depending on whether classification was done on full length or short variable region amplicons.

At the species level, we found that Parathaa’s default *specific* mode achieved mean F1 scores of 0.811 and 0.878 in the V1V2 and V4V5 regions, respectively, across the three holdout datasets. Naïve Bayes classification using exact sequence matching at the species level while still allowing for multiple matches (Naïve Bayes – Multi) had the best performance in comparison to not allowing multispecies matches (Naïve Bayes – Exact) achieving mean F1 scores of 0.845 and 0.650, respectively (Fig. 3A). Furthermore, across the holdout datasets, we found that Parathaa’s default specific settings found a higher proportion of correct ambiguous species when compared to Naïve Bayes – Multi on both V1V2 (Parathaa = 10.6%, NB – Multi = 2.3%) and V4V5 regions (Parathaa = 24%, NB – Multi = 19.2%) (Supplemental Fig. 1). Moreover, the high percentage of sequences assigned to multiple taxa, especially by Naïve Bayes Multi (which requires exact sequence matching), underscores the substantial sequence sharing among different species within the V4V5 region. This is often left unanalyzed by other methods.We also compared Parathaa to IDTAXA, a machine-learning based algorithm for taxonomic assignment of amplicons [30]. In the holdout dataset, we found Parathaa outperformed IDTAXA at the species level in both V1V2 and V4V5 variable regions.

To examine the benefits of Parathaa’s ability to assign ambiguous sequences beyond exact matching, we compared the number of correct and incorrect species assignments made by Parathaa and the tested Naïve Bayes classifiers within one of the holdout datasets. In the V1V2 region, Parathaa assigned a substantially larger number of sequences at the species level (*n* = 2792) compared to Naïve Bayes – Multi (*n* = 861), while also achieving a higher proportion of correct assignments (Parathaa = 87.7%, Naïve Bayes – Multi = 69.8%). Interestingly, among these assignments by Parathaa, 1129 were assigned to multiple taxa, whereas 292 were multiply assigned by Naïve Bayes – Multi (Fig. 3B). The higher number of multiple assignments made by Parathaa, along with its higher accuracy, highlights its stronger sensitivity while maintaining strong specificity within the V1V2 region compared to exact sequence matching. In the V4V5 region, both Parathaa and Naïve Bayes – Multi assigned a similar number of sequences (*n* = 4618 and 4286, respectively), with Parathaa achieving a slightly higher portion of correctly assigned labels (Parathaa = 83.3%, Naïve Bayes – Multi = 80.3%). In this region, Parathaa assigned 2723 ambiguous sequences, whereas Naïve Bayes – Multi identified 2179 sequences that matched exactly between multiple species (Fig. 3B). Parathaa thus consistently and correctly identifies more amplicon sequences with multiple equally valid taxonomic assignments, which are generally overlooked by other classifiers.

Parathaa’s default settings also achieved a higher F1 score in comparison to both Naïve Bayes – Exact and Naïve Bayes – Multi on all other short variable region datasets we tested (Supplemental Fig. 2) with the exception of the “genus outside of DB” dataset. Note, however, that this dataset is evaluated solely by the ability to not assign taxonomic labels to sequences that were from genera/species not in the seed database. Moreover, the differences in scores was minimal, with Naïve Bayes – Exact achieving an F1 score that was 0.005 higher in V1V2 and 0.004 higher in V4V5.

On full-length 16S rRNA gene data, we found that Parathaa’s default settings generally scored lower recall but higher precision than using Naïve Bayes species assignment without exact species matching (minboot = 0.8; Fig. 3A and Supplemental Figs 1 and 2). To address this, we introduced a second set of pre-configured parameters termed Parathaa *sensitive*, which widens the species assignment distance threshold. We found that Parathaa sensitive scored the highest mean F1 score in the holdout datasets (0.864) and identified 12.8% of sequences to be ambiguous at the species level. On the remaining synthetic datasets, Parathaa *sensitive* achieved the highest F1 score in comparison with the Naïve Bayes classifiers, again with the exception of the “genus outside of DB” dataset where Parathaa specific (F1 = 0.996) and Naïve Bayes – Exact (F1 = 0.999) performed the best (Supplemental Fig. 2). Comparing the number of sequences assigned by Parathaa sensitive and the tested Naïve Bayes classifier on one of the holdout datasets, we found that while Parathaa assigned a slightly smaller number of sequences at the species level overall (Parathaa = 5099, Naïve Bayes = 5345), it achieved a higher absolute number and percentage of correct assignments (Parathaa = 4101; 80%, Naïve Bayes = 3793; 71%). Of the correct Parathaa assignments, 1219 were multiply assigned, with the majority of such assignments receiving two (*n* = 907) or three (*n* = 209) assignments (Fig. 3B).

In addition to testing Parathaa and the RDP-based Naïve Bayes classifiers we also assessed whether varying parameters resulted in performance differences of one method over another. To do this, we ran both the RDP-based Naïve Bayes classifier over its complete allowed range of bootstrapping values (0–99) and Parathaa with increasing species multiplier thresholds up to two-fold (Fig. 3C). We found that, overall, Parathaa performed better than the Naïve Bayes classifier across the available range of parameter settings, almost always achieving better precision with similar recall. However, as previously shown, in some cases Naïve Bayes – Exact and Naïve Bayes – Multi did achieve higher precision at the cost of lower recall (Fig. 3C).

Finally, to assess Parathaa’s performance on a larger database, we trained Parathaa along with both the standard RDP Naïve Bayes classifier and the Naïve Bayes – Multi classifier with a 16S rRNA gene database built from GTDB R202 [31]. This allowed us to evaluate newly added (and thus held out) sequences from the latest R220 release, while also excluding any sequence found identically in the R202 release. The training database contained a total of 24 102 sequences after alignment and filtering out fragmented genes (<1400 base pairs in length) and spanned both bacterial and archaeal sequences. The evaluation set comprised 1783 further 16S rRNA gene sequences. Across all three tested regions (Full, V1V2, and V4V5), both Parathaa and the Naïve Bayes classifiers performed well, with Parathaa outperforming Naïve Bayes – Multi on the V4V5 region (F1 score: 0.616 Parathaa, 0.598 Naïve Bayes – Multi) but not on the V1V2 region (F1 score: 0.571 Parathaa, 0.594 Naïve Bayes – Multi) (Supplemental Fig. 3). All methods maintained generally higher precision than recall, with the exception of the standard RDP Naïve Bayes classifier on the full length dataset.

### Parathaa and Naïve Bayes – Multi exact species match perform similarly on a culture-based mock community

**Figure 4.**
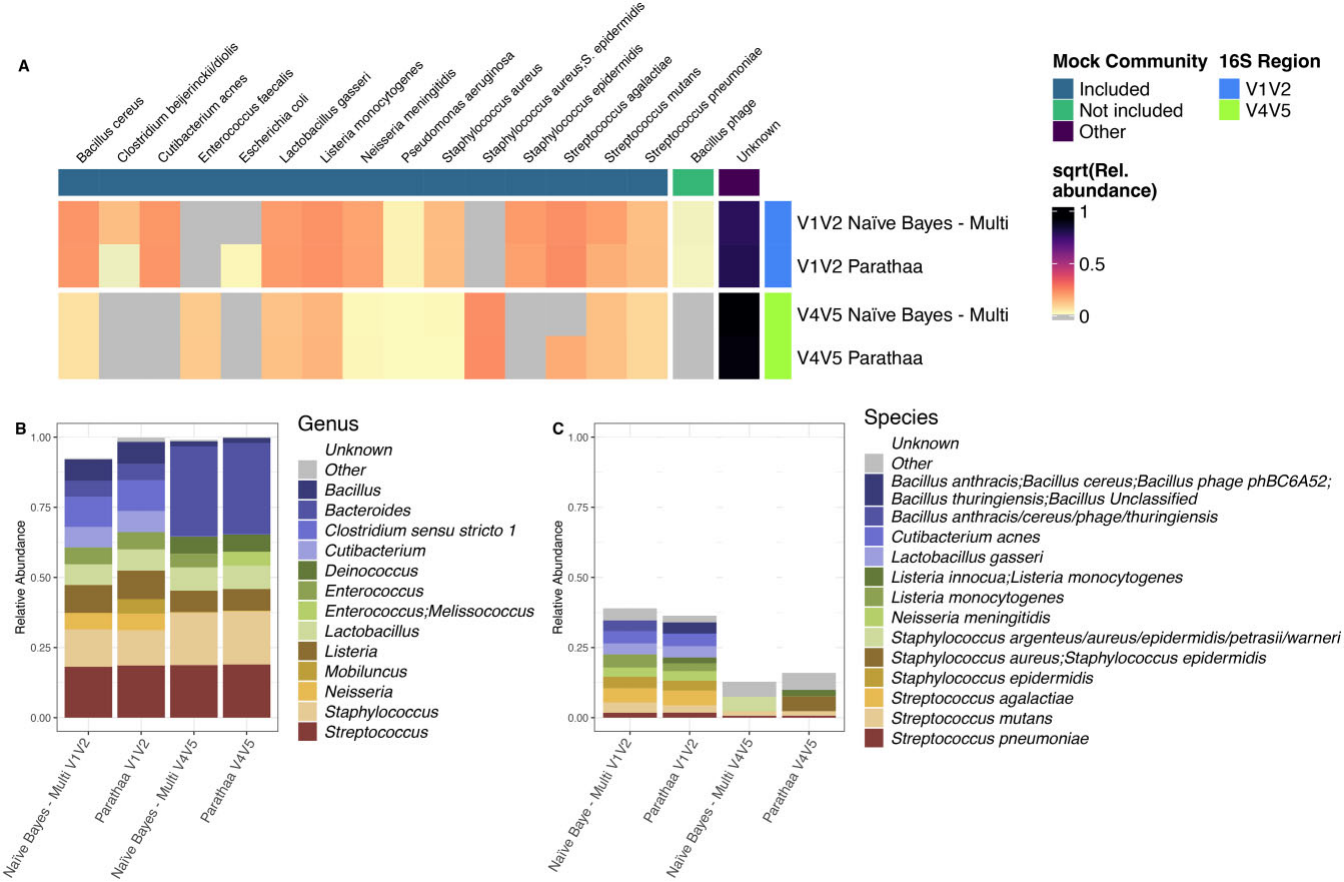
Performance of Parathaa and Naïve Bayes – Multi were largely comparable in a culture-based mock community. **(A)** Parathaa assigns reads to a similar number of species (V1V2) or more (V4V5) compared to Naïve Bayes – Multi. Heatmap comparing Naïve Bayes – Multi and Parathaa for taxonomic classification of a published cell-based mock communit [8]. The mock community is composed of DNA from 20 species in equal quantities (5% relative abundance). All species identified by either classifier corresponded with a species from within the mock community sample (“Included”). Ambiguous labels were cleaned to only show those that were included in the mock community. For a full list of ambiguous assignments see Supplemental Fig. 3. **(B)** Taxonomic profiles of mock community samples based on Naïve Bayes – Multi and Parathaa at the genus level. Genus-based profiles are generally similar between Naïve Bayes – Multi and Parathaa. **(C)** Taxonomic profiles of mock community samples based on Naïve Bayes – Multi and Parathaa at the species level.

As a second measure of Parathaa’s accuracy relative to a known ground truth, we applied Parathaa to a published mock community dataset of amplicon sequences that has previously been used to measure varying sources of bias in 16S rRNA gene sequencing data [8] (Fig. 4 and Supplemental Fig. 4). It consisted of 20 species whose DNA was present at 5% relative abundance each. From sequencing the V4V5 region, Naïve Bayes – Multi correctly identified ten species, while Parathaa identified eleven, of which three were strictly identified unambiguously, two had sequences that were identified both ambiguously and unambiguously, and six were identified as one of up to five ambiguous species (Fig. 4A and Supplemental Fig. 4). Interestingly, both Parathaa and Naïve Bayes – Multi labeled a sequence as “Bacillus Phage” despite being of 16S rRNA gene origin. Further inspection showed, however, that annotation errors within the SILVA database were the cause, as the source sequence within the ENA deposition was found to be *Bacillus cereus* ATCC 14579. Parathaa’s failure to detect nine of the species present using the V4V5 region was partly due to database effects (four were not represented in the SILVA seed database). In the V1V2 region, Naïve Bayes – Multi detected 12 included species, and Parathaa detected 13. Of these, Parathaa identified ten unambiguously, two as both ambiguous and unambiguous depending on the source sequence, and one as completely ambiguous at the species level (Fig 4A and Supplemental Fig. 4). Since amplicon data from small hypervariable regions are not always fully resolvable at the species level [32], full species detection was not expected from any method.

Examining overall relative abundance profiles, Parathaa classified a greater proportion of reads at the species level in the V4V5 region but not the V1V2 region data (Fig. 4C). At the genus level, taxonomic classifications by Parathaa are almost identical to those by Naïve Bayes – Multi, with the exception of *Mobiluncus*, in the V1V2 region; this is a misclassification by Parathaa of sequences from *Actinomyces*, a close relative (Fig. 4B). Parathaa also identified the sequences from the genus *Enterococcus* ambiguously relative to *Melissococcus*, while Naïve Bayes – Multi only identified them as being from *Enterococcus*. Parathaa’s ambiguity is arguably more correct, as *Melissococcus* currently only has one identified species within it and most likely represents an under-resolved genus that may actually belong within *Enterococcus* [33].

### Applying parathaa to real amplicon sequencing data

**Figure 5.**
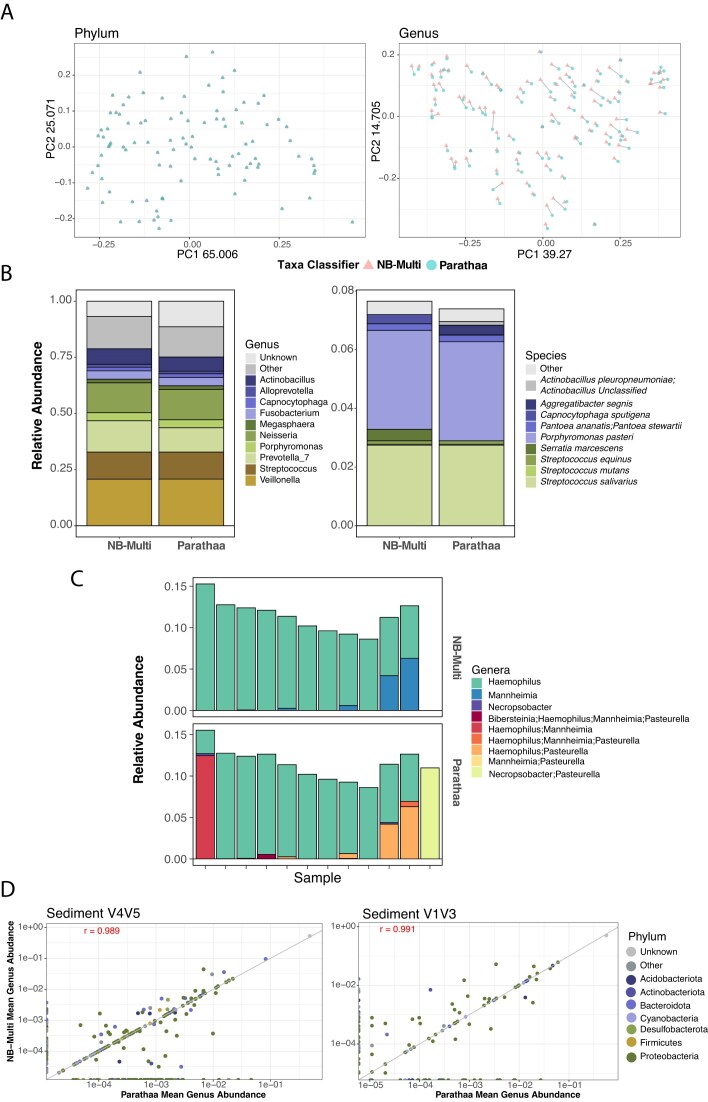
Parathaa and Naïve Bayes agree on saliva and sediment taxonomic profiles while also highlighting evidence of potentially polyphyletic genera. **(A)** Principal coordinates analysis for Bray–Curtis dissimilarities performed on 100 saliva microbiome taxonomic profile [17] from Naïve Bayes – Multi (NB – Multi) and Parathaa at either the phylum level or genus level after the removal of unassigned sequences. Lines connect points representing the same samples profiled by the two methods. **(B)** Average taxonomic profiles of 1214 oral saliva samples profiled by Naïve Bayes – Multi and Parathaa at the genus and species level. Taxa not in the top 11 most abundant were grouped into “Other”. **(C)** A subset of samples showing the relative abundances of sequences classified within Pasteurellaceae, a family that likely contains polyphyletic genera. Parathaa provides ambiguous genus assignments for many sequences, whereas Naïve Bayes – Multi assigns single genera despite having over 98% similarity to other genera in the 16S rRNA gene V4V5 sequenced region. **(D)** Correlation in the mean abundances of each genus detected by Naïve Bayes – Multi and Parathaa in 27 sediment samples using either V1V3 or V4V5 primers.

To assess how Parathaa performs on real-world amplicon data, we applied Parathaa to one human and one environmental study. The former comprised oral microbiomes of Atlantic Canadians using V4V5 16S rRNA gene sequencing [17] (Fig. 5A and B); the latter profiled microbial communities present in mine sediments using both V1V3 (Fig. 5C and Supplemental Fig. 5) and V4V5 sequencing (Fig. 5C and Supplemental Fig. 5). Our focus in this analysis was to broadly observe the percentage of reads classified at the genus and species levels, as well as to characterize systematic differences between Parathaa and Naïve Bayes – Multi at various taxonomic levels. As in the synthetic and mock community analyses, the SILVA seed database was used by both Parathaa and Naïve Bayes – Multi.

In the human oral microbiome dataset, profiles between tools generally agreed, especially at high taxonomic ranks (Fig. 5A), with the highest variation found at the genus level profiles (PERMANOVA R^2 ^= 0.023, *P *<.001; Fig. 5A). This was largely due to a greater percentage of sequences classified at the genus level by Naïve Bayes – Multi (mean 93.3%) than Parathaa (mean 88.6%; Fig. 5B). At the species level, both tools left the majority of ASVs unassigned (means Naïve Bayes – Multi 92.0%, Parathaa 92.6%). With both tools identifying a small subset of unique low abundance species. Of the 10 653 input ASVs, Parathaa assigned species to 120, while Naïve Bayes – Multi assigned species to 122. Naïve Bayes – Multi made 29 ambiguous species calls (3.2 potential species per assignment on average), and Parathaa made 61 (3.03 potential species per assignment on average). While these assignments made up the minority of classifications, the ability to give additional information about the taxonomic origin of these sequences can be critical in downstream applications.

To highlight this, we next decided to examine potentially ambiguous classifications within the family Pasteurellaceae, a highly abundant and prevalent human oral bacterial family [17, 34]. Pasteurellaceae includes 36 documented genera in NCBI, comprising commensal and pathogenic species found in animals and humans. Proposed phylogenies are under frequent revisions within the family, and it is likely that several of its genera within this family are polyphyletic, such as *Pasteurella, Mannheimia*, and *Haemophilus* [35]. In the human oral microbiome here, Pasteurellaceae make up an average of 7.9% of the relative abundance profiles from both Parathaa and Naïve Bayes – Multi (Fig. 5C). Within Pasteurellaceae, Parathaa assigns ambiguous genus designations to genera including *Bibersteinia, Haemophilus, Manheimia, Pasteurella*, and *Necropsobacter* (Fig. 5C). Naïve Bayes – Multi assigns (single) genera to many of these reads, despite high genetic similarity (>98% percent identity) of the V4V5 region to isolates from other Pasteruellaceae genera, confirmed by BLAST. Parathaa results highlight the phylogenetic ambiguity of this family in a way that would be missed by available classification tools, and in some cases, it is able to assign genus level classifications that were not previously possible.

We next compared Parathaa and Naïve Bayes – Multi’s performance on sediment samples as a representative under characterized environment. Unsurprisingly, we found that the proportion of unknown genera was higher compared to the oral dataset for both Naïve Bayes – Multi (V4V5 = 52.5%; V1V3 = 50.5%) and Parathaa (V4V5 = 48.6%; V1V3 = 56.4%; Supplemental Fig. 5). Despite this we found that mean genus abundances between tools were highly correlated in both V1V3 (Pearson, r = 0.991, *P *<.001) and V4V5 regions (Pearson, r = 0.989, *P *<.001) (Fig. 5C). Interestingly, in the V4V5 data, Parathaa was able to identify nine sequences as *Denitratisoma;Sulfuritalea*, making up on average 4.6% total relative abundance, while Naïve Bayes – Multi failed to assign any of these at the genus level (Supplemental Fig. 5). At the species level, both profilers failed to assign species in the V1V3 sediment dataset (Supplemental Fig. 4), but produced similar species level profiles at the V4V5 reads, with the exception of *Flavobacterium psychrolimnae* detected by Naïve Bayes – Multi but not Parathaa (Supplemental Fig. 5). Overall, Parathaa performed as expected across both variable regions within the sediment dataset producing similar profiles as Naïve Bayes – Mult (Fig. 5C and Supplemental Fig. 5).

## Discussion

A major limitation of amplicon sequencing is the ambiguity with which sequences, particularly short amplicons, may be assignable to multiple (or no) taxa; this has particularly impeded confident, accurate species-level taxonomic profiling. To address this, we developed Parathaa to model ambiguity among multiple possible taxonomic assignments, enabling investigators to retain more specific taxonomic information from their amplicon data with confidence. Parathaa assigns multiple taxonomic labels to a sequence when its true origin is quantifiably ambiguous with respect to its region-specific phylogenetic placement. Parathaa accomplishes this by (i) using primer-specific phylogenetic trees to identify distance thresholds for each taxonomic level that are specific to the sequenced region, (ii) defining single or one-to-many classifications for each taxonomic group defined by these optimized thresholds, and (iii) enabling taxonomic classification of novel sequences with unique or one-to-many labels as appropriate via phylogenetic placement.

Parathaa’s approach differs from widely used taxonomic classifiers, such as the Naïve Bayes classifiers implemented in both DADA2 [26] and QIIME2 [28] or IDTAXA [30], a machine learning approach implemented in the DECIPHER R package [25]. Both are well-studied in this application and often perform well. The main benefit of Parathaa’s approach is its guarantee on detecting all possible taxonomic assignments compatible with a given amplicon region’s taxonomy/phylogeny correspondence; as a result, it also “explains” such classifications using specific phylogenetically motivated parameters and placement. Indeed, Parathaa directly outputs reference trees and placement information for query reads, allowing users to easily assess the reasons for sequences’ taxonomic assignments (as in Fig. 1B). This contextualizes how each region-specific sequence relates both phylogenetically and taxonomically to other “nearby” reference sequences. Additionally, while a subset of other taxonomic classification software can assign multiple taxa to a given sequence, this information is generally given as a collection of top “hits” with corresponding percent sequence identity, without an indication of which of these is close enough to be a reasonable candidate at a given taxonomic level. While recent developments through tools such as unassigner [36] and DADA2’s option to output multiple species’ exact matches (through the use of a modified RDP Naïve Bayes classifier referenced here within as Naïve Bayes – Exact and Naïve Bayes – Multi) exist, the ambiguous nature of assignments for these tools are limited to the species level and do not directly incorporate the phylogenetic relationships or region-specific distances that Parathaa integrates.

As a result of these benefits, Parathaa achieved a higher F1 score compared to IDTAXA and various implementations of the RDP Naïve Bayes classifier across both subregions (V1V2 and V4V5) on four of five different synthetic dataset types, with minimal differences being found in the “genus outside of DB” dataset (see the ‘Methods’ section). Interestingly, these two subregions differed substantially with respect to the phylogenetic distance thresholds identified for the various taxonomic levels (Fig. 2C and D), with distances generally greater in the V1V2 than the V4V5 regions. This finding broadly agrees with previous work showing higher sequence entropy within the V1V2 region [32]. Thus, it is not surprising that Parathaa correctly assigned a higher number of ambiguous species in the V4V5 region (Fig. 3 and Supplemental Figs 1 and 2).

Parathaa and the Naïve Bayes classifier using exact sequence matching at the species level (Naïve Bayes – Multi) produced similar results on several sequenced datasets evaluated, especially on the mock community and at higher levels of taxonomy (e.g. phylum). In some cases, Parathaa was able to identify more species, such as in the mock dataset; in other cases, such as the V4V5 mine dataset, it agreed closely with Naïve Bayes – Multi. Interestingly, we did encounter a case in the V4V5 mine sediment dataset where Parathaa did not assign species level taxonomy to *F. psychrolimnae*, whereas Naïve Bayes – Multi exact matching did. Upon further inspection, this taxon has a neighboring species (*Flavobacterium limicola*) that differs by only two SNPs in its V4V5 region, which resulted in Parathaa not assigning a species level due to multiple placements above the specific species threshold. However, Parathaa was able to recover this assignment when using its sensitive mode (data not shown). Whether or not the sequences from this study were from *F. psychrolimnae* is unknown, but it is possible that even exact matches could result in incorrect taxonomy due to imperfect systematics relative to phylogeny, a problem that Parathaa directly models in its primer-specific phylogenetic tree and by setting specific boundaries of phylogenetic distance on each taxonomic level. Aside from this Parathaa was also able to identify a large portion of V4V5 sediment sequences as ambiguous between the genera *Denitratisoma* or *Sulfuritalea* an assignment that would have been missed using Naïve Bayes – Multi. Moreover, Parathaa was able to correctly model the polyphyletic nature of the *Pasteruellacea* family [35], commonly present in the human oral microbiome [34]. Indeed, Parathaa was able to assign multiple sequences with ambiguous genera when the V4V5 sequenced region showed above 98% similarity, whereas Naïve Bayes – Multi either assigned a single genus or no genus at all (Fig. 5C). This highlights Parathaa’s ability to highlight cases where sequence similarity within a specific region disagrees with current taxonomic labels.

During the development of Parathaa, we noticed that the “optimal” species threshold determined by its algorithm (Parathaa *sensitive*) often achieved the highest F1 score but had reduced precision when compared to some implementations of the Naïve Bayes classifier we tested (Fig. 3 and Supplemental Fig. 1). As in the example above, this proved to be frequently driven by disagreements between taxonomy and phylogeny; it is likely that in at least some cases, Parathaa’s “incorrect” assignments are more accurate than those annotated to deposited sequences. To address this, we included a *specific* species assignment mode where Parathaa reduces the optimally identified species threshold by 10-fold to prioritize higher precision species level classifications while still maintaining a higher recall than both Naïve Bayes – Exact and Naïve Bayes – Multi (Fig. 3). While this resulted in slightly lower F1 scores, it allows users to focus only on species-level assignments that are consistent with both pre-existing systematics and with phylogeny, which we provide as the default in Parathaa. However, it should be noted that in some cases, particularly when working with full-length amplicons, the default “specific” mode in Parathaa may be overly conservative. In such instances, users may prefer to utilize the “sensitive” mode, which enables broader taxonomic classifications that reflect the phylogenetic signal present in the sequenced region. However, it is important to note that a subset of these classifications may differ from current systematics or from existing annotations in other reference databases.

Parathaa thus represents a novel method for taxonomic classification of amplicon sequences, based on region-specific phylogeny and with explicit guarantees on ambiguous assignments and taxonomy/phylogeny relationships. It performed with comparable accuracy to gold standard methods on synthetic (on both Silva and GTDB databases), mock community, and real-world data. However, a limitation worth mentioning is one of Parathaa’s default databases, the “seed” database provided by mothur, is a small subset of the available 16S rRNA gene sequences in the full/NR99 SILVA database [18] or in other 16S rRNA gene databases [37, 38]. We chose this as Parathaa’s default as it is of a manageable size for the dependencies of its constituent components, particularly *pplacer*, which is not designed to work with ultra-large reference trees [39]. Due to this restriction on tree size, Parathaa will have lower theoretical limits on database size in comparison to tools such as the RDP Naïve Bayes classifier. This has limited impact in practice; we include, for example larger databases using GTDB R220 (V1V2, V4V5, V1V3, and full length). This database contains over 40 000 small subunit sequences and is not noticeably computationally constrained (Supplemental Fig. 3). In a future update, we hope to explore larger databases by evaluating accelerated implementation for sequence placement in ultra-large phylogenetic trees [39–41].

There is also an opportunity to explore Parathaa’s unique ability to identify phylogenetic distance thresholds for taxonomic groups to curate large reference databases and identify potentially mis-annotated sequences or polyphyletic taxonomies that are inconsistent with 16S rRNA gene phylogenies. In addition, there is an added value to investigate a more dynamic way to identify distance thresholds across phylogenetic trees. This may help tune Parathaa to better match the phylogenetic landscape of particular taxonomic groups. Finally, we would also highlight that while benchmarking for this manuscript was only accomplished on full length, V1V2, and V4V5 16S rRNA gene regions, Parathaa’s implementation lends itself to be agnostic of database and amplicon choice. As such, it is well positioned to be benchmarked and used with other amplicon databases such as the Unite [42] database for ITS sequences or other rRNA gene databases such as Greengenes2 [43].

## Conclusions

Here, we introduced Parathaa, a novel method that uses a region-specific phylogenetic approach to perform taxonomic classification for amplicon sequences while retaining taxon- and region-specific ambiguity via one-to-many assignments. Parathaa accomplishes this while maintaining similar or better performance than state-of-the-art classification tools as implemented in IDTAXA or various iterations of the RDP Naïve Bayes classifier. In addition, Parathaa produces more interpretable classifications through taxonomic placement and justification using neighboring sequences within primer-specific phylogenetic trees. Parathaa’s implementation is available open source via PyPI and GitHub (https://huttenhower.sph.harvard.edu/parathaa) and includes pre-made databases for full length, V1V2, V1V3, V4V5, and V3V4 16S rRNA gene subregions.

## Supplementary Material

gkaf1291_Supplemental_File

## Data Availability

Parathaa and its installation instructions are available in GitHub as linked at: https://huttenhower.sph.harvard.edu/parathaa. A completely reproducible AnADAMA2 workflow for all figures and analysis in this paper is also available in an additional GitHub repository linked there, as is documentation, a tutorial, and demonstration data. Raw sequence data from the sediment dataset is available on BioProject (https://www.ncbi.nlm.nih.gov/bioproject) under accession number PRJNA1167286.
