# Supplementary material for: Capturing sequence ambiguity among taxa in a primer-specific manner to improve taxonomic classification of amplicon sequencing
Source: Nucleic Acids Res. 2025 Nov 29;53(22):gkaf1291. doi: 10.1093/nar/gkaf1291 (PMC12663084; doi:10.1093/nar/gkaf1291)
Supplement: gkaf1291_Supplemental_File [file gkaf1291_supplemental_file.pdf]

Supplemental Figures

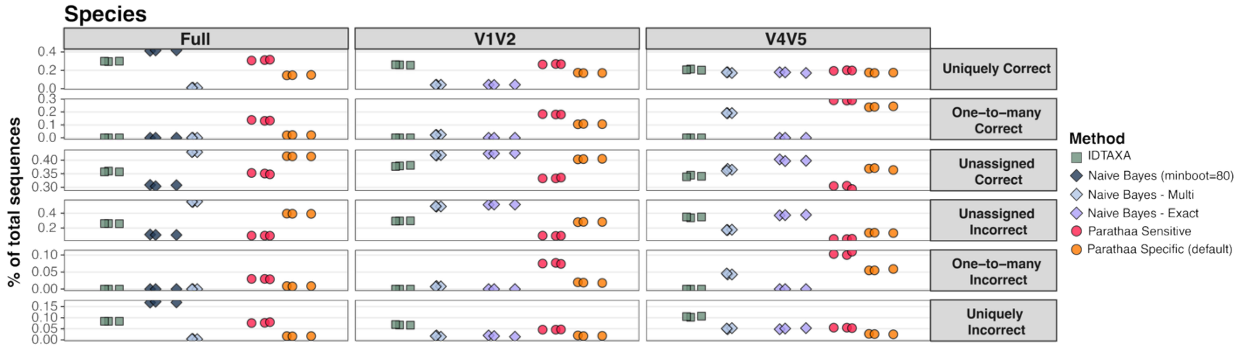

Supplemental Figure 1: Sequence classifications for various amplicon classifiers across three separate synthetic holdout datasets. Parathaa's performance was compared to Naïve Bayes classifiers on both full-length and short region 16S rRNA gene sequences from three separate holdout datasets (n=10,000) from the Silva v138 database. For short-region sequences (V1V2, V4V5), we evaluated Parathaa against two Naïve Bayes implementations: (1) a classifier requiring exact matches at the species level (Naïve Bayes – Exact), and (2) a classifier requiring exact species-level matches but permitting multiple species assignments for a single input sequence (Naïve Bayes – Multi). For full-length sequences, Parathaa was compared to either Naïve Bayes - Multi or a standard Naïve Bayes classifier with a minimum bootstrap value of 80 (Naïve Bayes (minboot=80)). The six rows show the % of total input sequences that were either uniquely correct, one-to-many correct, unassigned correct, unassigned incorrect, one-to-many incorrect, or uniquely incorrect.

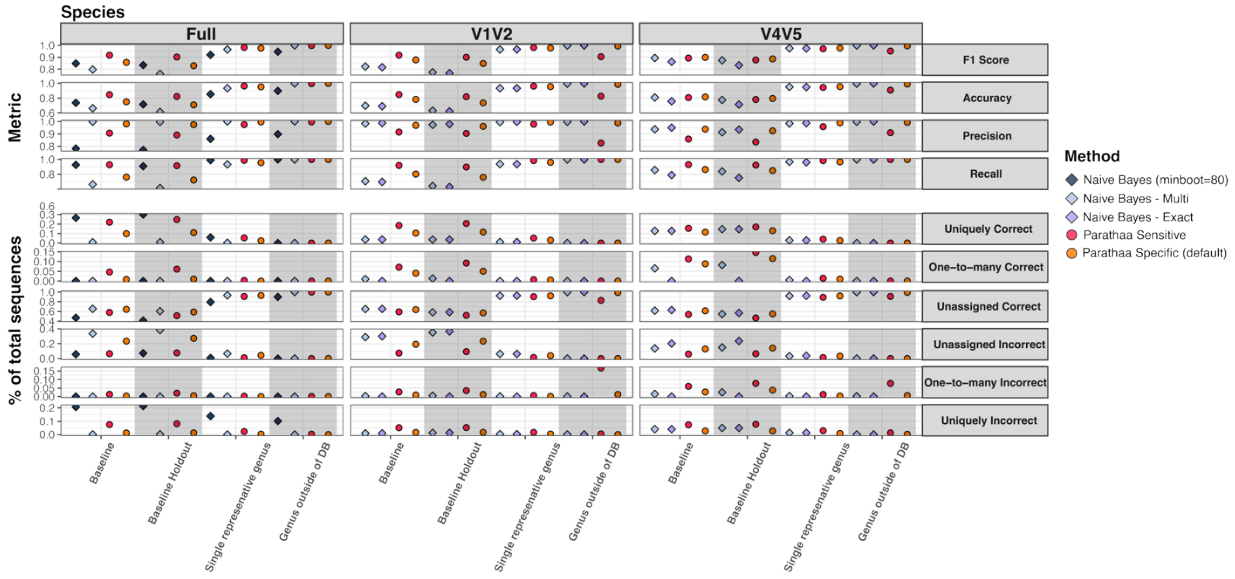

Supplemental Figure 2: Full performance metrics of Parathaa, and Naïve Bayes classifiers at the Species level across four different synthetic datasets. Parathaa's performance was compared to Naïve Bayes classifiers on both full-length and short region 16S rRNA gene sequences on four different synthetic datasets (Methods). For short-region sequences (V1V2, V4V5), we evaluated Parathaa against two Naïve Bayes implementations: (1) a classifier requiring exact matches at the species level (Naïve Bayes – Exact), and (2) a classifier requiring exact species-level matches but permitting multiple species assignments for a single input sequence (Naïve Bayes – Multi). For full-length sequences, Parathaa was compared to either Naïve Bayes - Multi or a standard Naïve Bayes classifier with a minimum bootstrap value of 80 (Naïve Bayes (minboot=80)). The first four rows show the classifiers F1 score, Accuracy, Precision, and recall across the tested synthetic datasets. The bottom six rows show the % of total input sequences that were either uniquely correct, one-to-many correct, unassigned correct, unassigned incorrect, one-to-many incorrect, or uniquely incorrect.

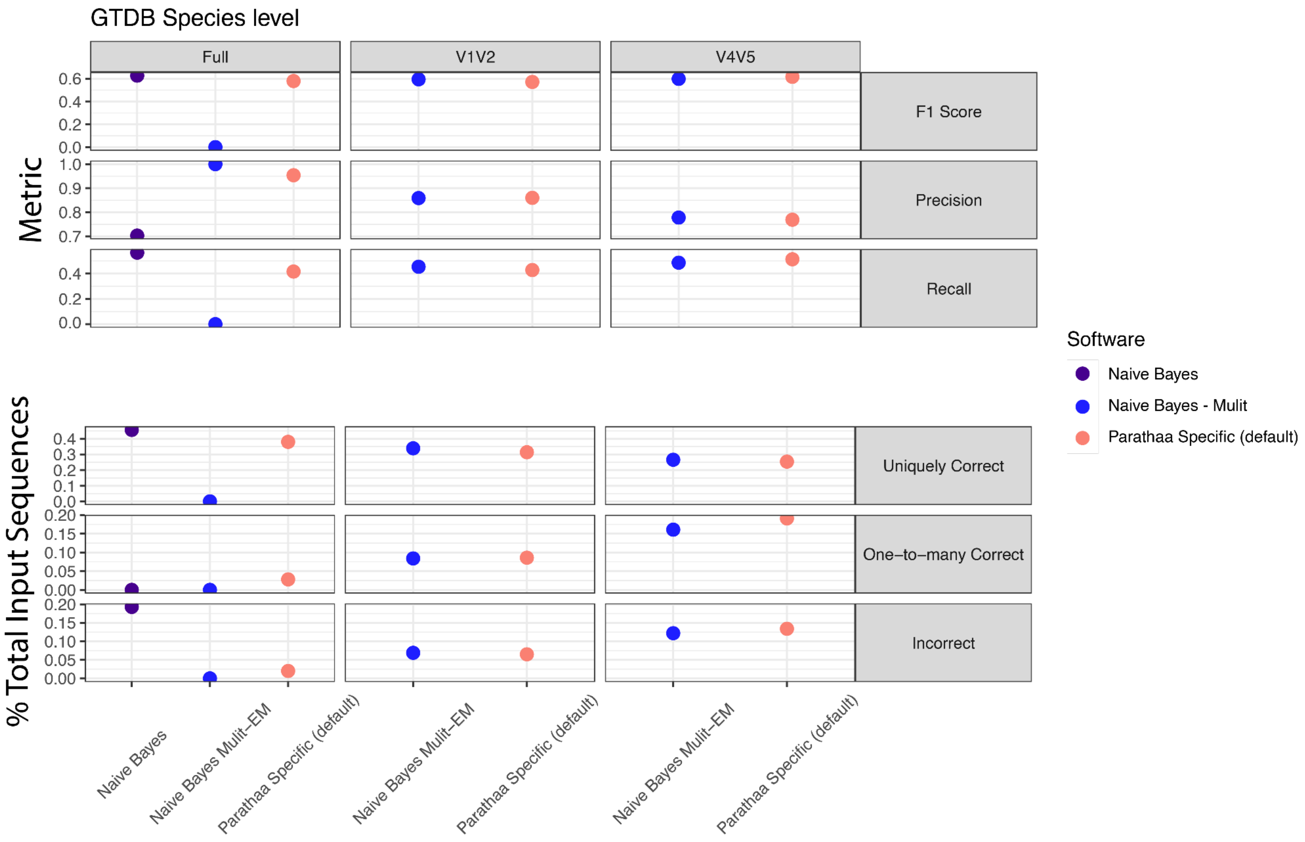

Supplemental Figure 3: Parathaa and Naïve Bayes species level performance on GTDB R202 using historical holdouts from GTDB R220. Parathaa and Naïve Bayes classifiers either requiring exact species matching (Naïve Bayes - Multi) or not (Naïve Bayes) - were trained on the 16S rRNA gene database from GTDB R202. Newly added sequences (n=1,783) were then extracted from GTDB R220 that were from species represented in R202 but not exact matches to any sequence in R202. The top three plots show the performance values for either the F1 score, Precision, or Recall. The bottom three plots show the percent of total input sequences that were either uniquely correct, one-to-many correct, or incorrect.

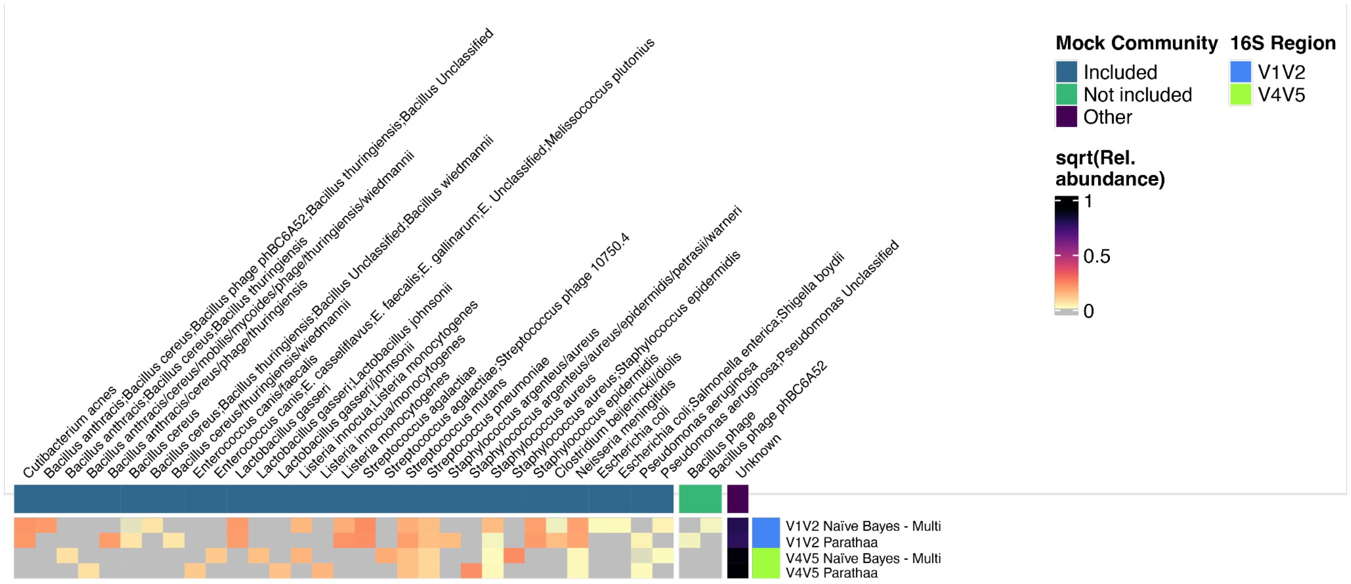

Supplemental Figure 4: Parathaa and Naïve Bayes - Multi species level assignments to mock community data. Heatmap comparing Naïve Bayes and Parathaa species level taxonomic classification of a published cell-based mock community[8]. The mock community is composed of DNA from 20 species in equal quantities (5% relative abundance). Species that were expected were labeled as “included”. Species with multiple species assignments by either Naïve Bayes - Multi or Parathaa were considered included if at least one species was expected in the mock community. Taxon that were supported by less than 30 reads were filtered out.

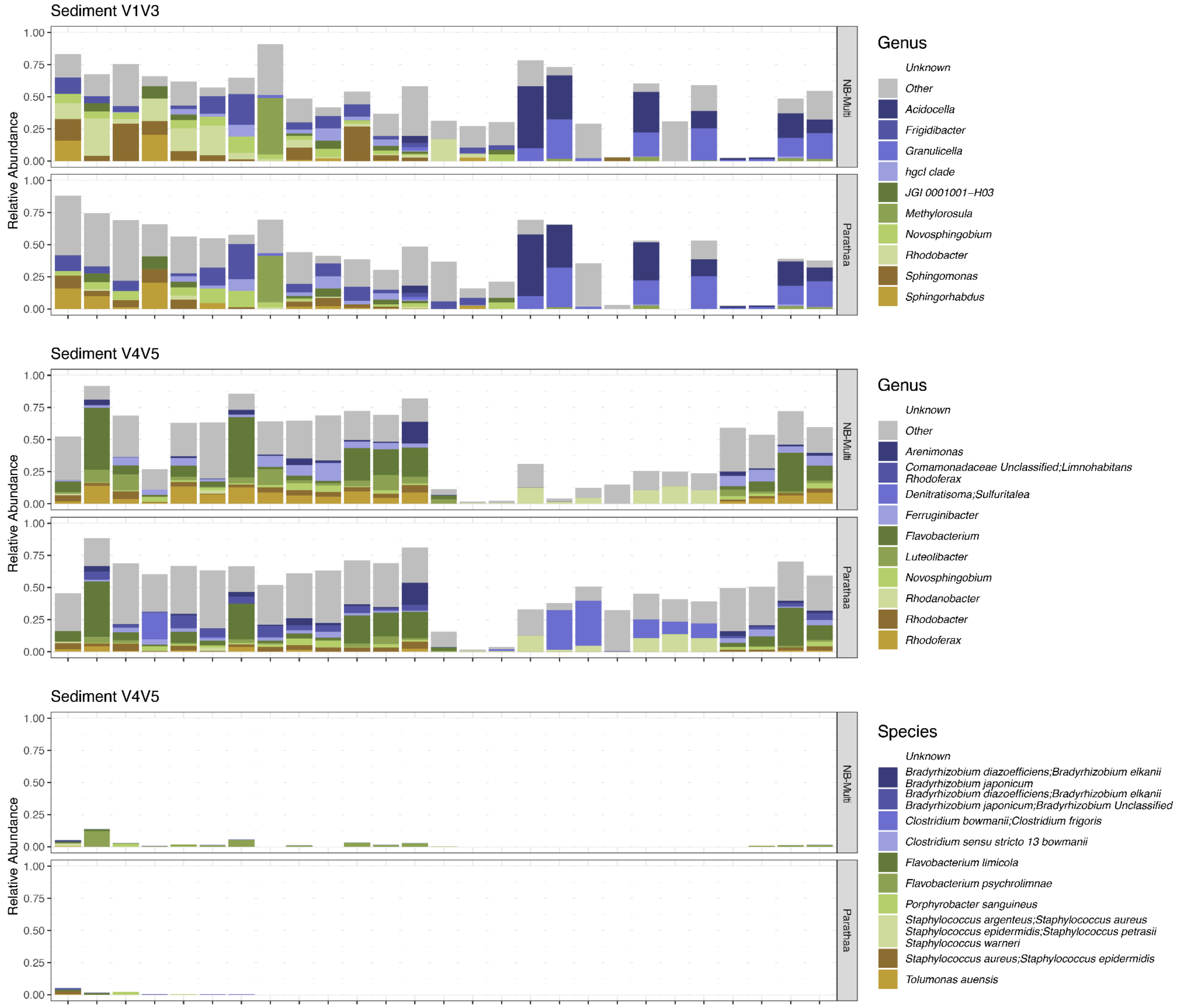

Supplemental Figure 5: Taxonomic profiles of sediment V1V3 and V4V5 data. Genus and species level profiles from 27 sediment samples using both V1V3 and V4V5 sequencing. Both Naïve Bayes - Multi and Parathaa failed to assign any species in V1V3 data leaving only V4V5 species level profiles.
